# Time of Lactation and Maternal Fucosyltransferase Genetic Polymorphisms Determine the Variability in Human Milk Oligosaccharides

**DOI:** 10.3389/fnut.2020.574459

**Published:** 2020-10-29

**Authors:** Gregory Lefebvre, Maya Shevlyakova, Aline Charpagne, Julien Marquis, Mandy Vogel, Toralf Kirsten, Wieland Kiess, Sean Austin, Norbert Sprenger, Aristea Binia

**Affiliations:** ^1^Société des Produits Nestlé SA-Nestlé Research, Ecole Polytechnique Fédéral de Lausanne Innovation Park, Lausanne, Switzerland; ^2^Société des Produits Nestlé SA-Nestlé Research, Lausanne, Switzerland; ^3^Hospital for Children and Adolescents and Center of Paediatrc Research, LIFE Forschungszentrum für Zivilisationserkrankungen, Medizinische Fakultät, University of Leipzig, Leipzig, Deutschland

**Keywords:** breast milk, HMO, FUT2, oligosaccharides, FUT3

## Abstract

**Rationale:** Human milk oligosaccharides (HMOs) vary among mothers and genetic factors contribute to this variability. We assessed changes in HMO concentrations during the first year of lactation and the relationship with *FUT2* Secretor group and *FUT3* Lewis group defining genetic polymorphisms.

**Methods:** Milk samples were collected from lactating mothers participating in the LIFE Child cohort in Leipzig, Germany. The concentrations of 24 HMOs in milk samples collected at 3 months (*N* = 156), 6 months (*N* = 122), and 12 months (*N* = 28) were measured using liquid chromatography. Concentrations of HMOs were compared at all time-points and were tested for their associations with *FUT2* and *FUT3* genetic variations by sPLS regression.

**Results:** *FUT2* SNP rs601338 was found to predominantly define the Secretor status Se-: 11.8% and it was highly correlated with 2′-fucosyllactose (2′FL, *p* < 0.001) and lacto-N-fucosylpentaose-I (LNFP-I, *p* < 0.001). *FUT3* SNPs rs28362459 and rs812936 were found to define Lewis status (Le-: 5.9%) and correlated with lacto-N-fucosylpentaose-II (LNFP-II, *p* < 0.001). A polygenic score predicted the abundance of 2′FL levels within Secretors' milk (adj. *R*^2^ = 0.58, *p* < 0.001). Mean concentrations of most of the individual HMOs, as well as the sums of the measured HMOs, the fucosylated HMOs, and the neutral HMOs were lower at 6 and 12 months compared to 3 months (*p* < 0.001).

**Conclusions:** Secretor and Lewis status defined by specific *FUT2* and *FUT3* SNPs are confirmed to be good proxies for specific individual HMOs and milk group variabilities. The polygenic score developed here is an opportunity for clinicians to predict 2′FL levels in milk of future mothers. These results show opportunities to strengthen our understanding of factors controlling FUT2 and FUT3 functionality, the temporal changes and variability of HMO composition during lactation and eventually their significance for infant development.

## Introduction

Human milk oligosaccharides (HMOs) are unconjugated glycans found in human milk and they are composed of the monosaccharides glucose, galactose, fucose, *N*-acetylglucosamine and *N*-acetylneuraminic acid, the main form of sialic acid in humans. They represent the third most abundant solid component of the human milk (5 to 20 g/L) after lactose and lipids (1–4). More than 200 HMOs have been separated, and around 150 of these have been characterized (5). Although non-digestible oligosaccharides are found in most mammalian milks (6), the oligosaccharide profile in human milk is of unique diversity (7, 8).

In general, the concentration of HMOs decreases during lactation, although only few studies have gone beyond the first 6 months of lactation (9–11). Generally, the sum of quantified HMOs is highest in colostrum secreted by the mammary gland during the first few days after birth and decreases thereafter (1, 12, 13). Some HMOs have a specific concentration trajectory over time (1) with most decreasing and few increasing in concentration suggesting the presence of regulatory mechanisms that control their temporal variation.

Several fucosyltransferases (FUT) are suggested to be involved in HMO synthesis (14, 15). Of these, FUT2 and FUT3 enzymes catalyze the α1,2-, and α1,3/4- transfer, respectively, of a fucose group to the core oligosaccharide structure (16). Genetic variations on *FUT2* and *FUT3* genes can lead to enzyme inactivation resulting from a premature stop of protein synthesis or a production of a truncated protein with very low activity (17, 18). Mothers who carry these inactivating mutations on *FUT2* are referred to as non-Secretors, as they lack the major α-1,2-fucosylated glycans in body secretions like saliva (19). Based on FUT2 enzyme specificity, the presence or absence of HMOs like 2′-fucosyllactose (2′FL) and lacto-N-fucopentaose-I (LNFP-I) in breast milk is generally discussed to be due to genetic polymorphisms in *FUT2* (18, 20). Phenotypic Secretor status determination and enzyme characterization confirmed this assumption (21). Similarly, milk samples derived from mothers with inactive FUT3 due to genetic variations are referred to as Lewis negative, as opposed to Lewis positive when FUT3 is active. Again, based on enzyme specificity, polymorphisms in *FUT3* are expected to be related to the α-1, 3/4-fucosylated HMOs, like lacto-N-fucopentaose II (LNFP-II) (14).

Milk samples can be assigned to one of four milk groups depending on combinations of presence or absence of HMOs containing α-1,2-linked fucose residues (2′FL, LNFP-I, Secretor-specific) and α-1,4-linked fucose residues (LNFP-II, Lewis-specific) with expected presence or absence of the respective FUT2 and FUT3 enzyme activities (22, 23). Following this, milk group 1 corresponds to Secretor and Lewis positive (Se+Le+), milk group 2 corresponds to Non-Secretor and Lewis positive (Se-Le+), milk group 3 corresponds to Secretor and Lewis negative (Se+Le-) and milk group 4 to Non-Secretor and Lewis negative (Se-Le-) profile. Each milk group appears to bear a specific HMO profile with a characteristic trajectory over time defined by the presence of active fucosyltransferases and substrate availability (1). In addition, previous studies have also shown that Secretor status can affect not only the production of specific fucosylated HMOs but also the overall concentration of HMOs with non-Secretors apparently having a significantly lower concentration of total measured HMOs (13, 24).

Most of the *FUT2* and *FUT3* genetic variations are single nucleotide polymorphisms (SNP) characterized by a replacement of a single nucleotide. Some of them lead to a replacement of an amino acid or an early termination of the protein synthesis (functional SNPs).The most studied example is *FUT2* SNP rs601338 known to be the predominant variant leading to the non-Secretor phenotype in the European population (17, 25). It results in a stop codon that leads to premature termination of protein expression and complete abolition of the enzymatic activity (26). There is, however, no current study in the literature systematically investigating the effect of functional *FUT2* and *FUT3* SNPs on the concentration of individual and total measured HMOs in milk.

We sought to assess the impact of *FUT2* and *FUT3* SNPs on concentrations of HMOs in a cohort of 156 lactating women from Leipzig, Germany at 3, 6 and 12 months, aiming to establish a link between individual genetic variations and specific HMO profiles over the first year of lactation. In addition, we aimed to test how genetic variation in *FUT2* and *FUT3* combined can predict milk groups, as well as concentrations of individual HMOs.

## Materials and Methods

### Study Population

LIFE Child is a longitudinal epidemiological childhood cohort study initiated in 2011 in Leipzig, Germany. The study aims to follow children from pregnancy into young adulthood and determine risk and resilience factors for healthy development. The study is described in detail elsewhere (27, 28). In the child's first year of life, visits are scheduled at the age of 3, 6, and 12 months of life. Between 2011 and 2015, 156 lactating mothers visited the study center providing 156 milk samples at the 3-months visit, 122 at the 6-months visit, and 28 at the 12-months visit. Mothers were aged between 23 and 42 years at the child's birth. Blood samples from the mothers were collected between 2011 and 2015. DNA was isolated within 48 h after blood withdrawal on the QIAGEN Autopure LS platform using chemistry by Qiagen and Stratec Molecular and DNA samples were stored at −80°C in the LIFE-Biobank until usage.

### Sequencing Samples

DNA quantification was performed using the Picogreen (Life Technologies) ultra-sensitive fluorescent nucleic acid stain for quantifying double-stranded DNA on all samples. For a representative subset of samples, DNA integrity was validated with the TapeStation (Agilent) Genomic DNA ScreenTape.

The entire *FUT2* and *FUT3* coding sequences and part of 3′ and 5′ untranslated regions were PCR amplified for 30 cycles using the Kapa HiFi (Roche), starting from 50 ng DNA. PCR primer sequences were ACACACCCACACTATGCCTG (FUT2-Fw), AAGAGAGATGGGTCCTGCTC (FUT2-Re), CCCGGAGCTTTGGTAAGCAG (FUT3-Fw), and GAGGGTTGGCCACAAAGGAC (FUT3-Re). The same melting temperature of 60°C was used for both amplifications. A positive control (DNA from HapMap NA18523) and a No Template Control (water) were included on each PCR plate. The quality and quantity of each *FUT2* and *FUT3* PCR were checked by gel electrophoresis using the LabChip GX Touch (Perkin Elmer).

After purification on Ampure beads (Beckman) at a 1.8X ratio, sequencing libraries were prepared from the amplicons using the Nextera XT kit (Illumina) strictly following manufacturer's recommendations. Libraries were quantified with Picogreen (Life Technologies) and their size pattern validated with Fragment Analyzer (AATI). Sequencing was performed as a paired end 250 cycles run with the MiSeq Reagent Kits v2 (Illumina). The dataset is available at Sequence Read Archive (SRA) under project ID PRJNA643141.

### Calling Genetic Variants

Variant calling was performed with the software FreeBayes Garrison and Marth 2012 using default parameters. In order to perform the computation of such a large dataset, a script to parallelise the computation has been implemented and SNP calling has been split by batch of 200bp-long region. The resulting *vcf* files were then post-processed with the *plink* software v1.9 for quality control (QC) purposes and recoding. The quality check was performed in 3 steps. First, samples with more than 5% of missing genotypes were filtered out (–mind 0.05) and 1 sample was removed due to missing genotype data. Then, variants missing in more than 5% of the samples were filtered out (–geno 0.05): 24 variants were removed due to missing genotype data. Finally, variants with minor allele frequency (MAF: the frequency of the rare polymorphism in the population) below 1% computed on cohort data were filtered out (–maf 0.01), 2435 variants were removed due to minor allele threshold. Finally, 23 SNPs and 152 samples passed the filters and the QC.

### Determining Secretor, Lewis Status and Milk Groups

The classification of individuals in each Le/Se type was based on the genotype of functional SNPs; for Lewis (Le) type: rs3745635, rs28362459, rs3894326, rs812936 and for Secretor (Se) type: rs601338, rs1047781, and rs200157007, respectively. If the minor allele was found in homozygote form for at least one SNP, individuals were classified according to group definition, meaning Lewis negative (Le–) and non-Secretor (Se–), respectively. Milk groups were defined as previously described: milk group 1 corresponds to Secretor and Lewis positive (Se+Le+), milk group 2 corresponds to Non-Secretor and Lewis positive (Se–Le+), milk group 3 corresponds to Secretor and Lewis negative (Se+Le–) and milk group 4 to Non-Secretor and Lewis negative (Se–Le–).

### Determination of HMOs

HMOs were analyzed according to the method of Austin & Benet (29). Quantification of 2′FL, 3FL, 3′SL, 6′SL, A-tetra, LNT, LNnT, and LNFP-I was performed against genuine standards purchased from Elicityl (Crolles, France). All other HMOs were quantified against maltotriose (Sigma, Buchs, Switzerland) as a surrogate standard assuming equimolar response factors.

### Comparison of HMO Concentrations

We tested for differences in HMO concentrations by group of maternal genotypes for each SNP determining Secretor and Lewis status as outlined above. We used a Mann-Whitney test for non-matched non-parametric data with significant level threshold set to 0.0001. We performed pairwise comparisons and only retained the significant ones.

We calculated correlation coefficents between the different HMOs based on their concentrations in milk after log transformation by applying Pearson's product moment correlation coefficient and adjusted for multiple comparisons by controlling the false discovery rate.

### Dynamics of HMOs Over Time of Lactation

HMOs were combined in to 3 categories “fucosylated,” containing all the measured fucosylated HMOs, “sialylated,” containing all the measured sialylated HMOs, and “core” containing the core non-fucosylated HMOs LNT, LNnT, LNH, Hex2HexNAc4, as well as 3′GL and 6′GL, although the latter two are not strictly core structures. Dynamic changes for categories of HMOs (core, fucosylated, and sialylated HMOs) were assessed by fitting quantile regression (30) (tau = 0.5) of log concentrations with time-points in months. Confidence intervals for the estimated parameters are based on inversion of a rank test.

Clusters of HMOs and their boundaries were determined with multiscale bootstrap resampling of the correlation values with complete distance and *p*-value threshold set to 0.05.

### Genetic Markers of Milk Groups

In order to select the best SNP to predict the milk group the mother belonged to, we first performed a Sparse Partial Least Square regression (sPLS) on both the concentration of individual HMOs and the SNP matrices. This resulted in a first selection of 14 SNPs among the 24 measured HMOs. Then in a second step, we performed prediction modeling by testing 46 different models splitting the dataset in training and testing datasets. The Multi-Layer Perceptron (MLP) model performed best with highest accuracy of 0.978, representing the proportion of correct predictions to the total number of predictions. Although other models performed similarly, the MLP has been selected for its ease of interpretation compared to the others. Eventually, combinations of the weights in the network (31) were used to estimate the importance of the variables in the model.

### Polygenic Prediction Score for 2′FL in Milk of Secretor Mothers

A Generalized Linear Model (GLM) in both directions was used in a stepwise approach to select the individual and combinations of SNPs that best predict 2′FL concentration in breast milk. Each SNP in the model was encoded by 0, 1, or 2 for homozygous major allele, heterozygous and homozygous minor allele, respectively. The selected model was trained on a training dataset 200 repeats of 40-fold cross validation. The evaluation of the model was performed on an independent test dataset. The selected SNPs were included in an algorithm to compute a genetic score. The genetic score was defined as the sum of the alleles for the SNPs selected in the model. Then we regressed the 2′FL concentration with the genetic score on a training set to define the prediction model. Finally, we tested the prediction on an independent test dataset. We showed the genetic score was able to predict the concentration of 2'FL with an adjusted *R*-Square of 0.58.

## Results

### Concentrations of HMOs During the First Year of Lactation

**Figure 1:**
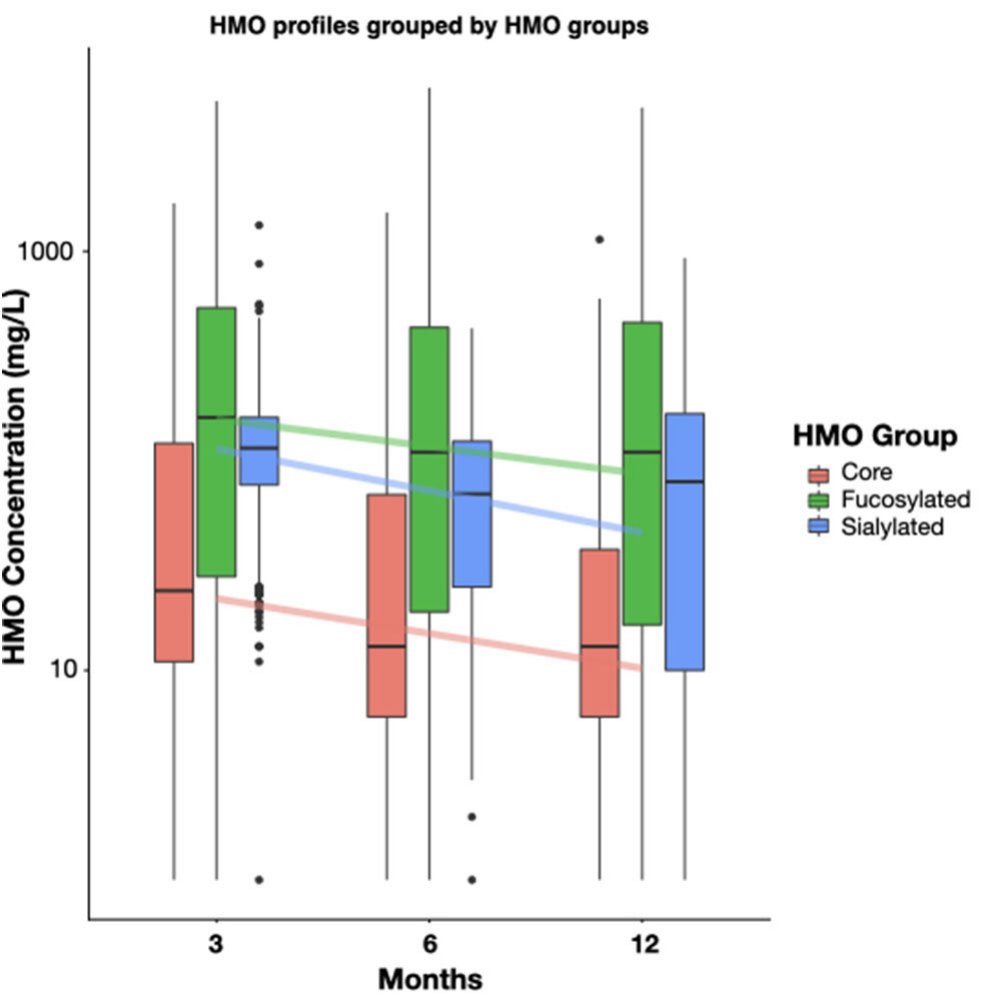
Dynamics of HMO concentration by the 3 main HMO classes, (i) core, (ii) fucosylated, and (iii) sialylated HMOs, between 3 and 12 months of lactation. The core class represents the sum of; LNT, LNnT, Hex4HexNAc2, LNH, 3′GL, 6′GL; The fucosylated class represents the sum of: A-tetra, 2′FL, LDFT, 3FL, LNFP-I, LNFP-II, LNFP-III, LNFP-V, LNnFP-V, LNnDFH,LNDFH-I, DFLNHa, MFLNH-III; The sialylated class represents the sum of: 3′SL, 6′SL, LSTb, LSTc, DSLNT. Boxplots depict median with interquartile ranges and whiskers indicate the minimum and maximum values. For all classes the 3 months to 6 months and the 6 months to 12 months changes were significant by non-parametric *t*-test (*p* < 0.05). Concentrations are shown as mg/L.

HMO concentrations have been measured in breast milk during the first year of lactation at 3, 6 and 12 months of infant age. HMOs were grouped as core structures such as LNT, LnNT, fucosylated structures such as 2′FL, 3FL, and sialylated structures such as 3′SL, 6′SL, (Figure 1, Supplementary Table 1). The summed concentrations of HMOs decreased from the 3^rd^ to the 12^th^ month of lactation, (Figure 1, Core: −0.070, *q* = 0.005, Fucosylated: −0.073, *q* < 0.001, Sialylated: −0.122, *q* < 0.001). This remained true for most individual HMOs regardless of the milk group of the mother (Supplementary Table 2). However, 3FL concentrations in milk from mothers with Se+/Le+ status significantly increased during the first year of lactation (Table 1).

**Table 1:** Dynamics of individual HMO concentrations during the 3 to-12-month lactation period.

**HMO**	**Milk group**	**Coefficient**	***q*-value <0.1**
2'FL	Se+/Le+	−0.167	0.051
3FL	Se+/Le+	0.204	0.087
6'SL	Se–/Le+	−0.960	0.002
6'SL	Se+/Le–	−0.888	0.059
6'SL	Se+/Le+	−1.049	<0.001
DFLNHa	Se+/Le+	−0.777	<0.001
LNDFH-I	Se+/Le+	−0.212	0.065
LNFP-I	Se+/Le+	−0.408	<0.001
LNFP-III	Se+/Le+	−0.203	<0.001
LNH	Se+/Le–	−0.571	0.003
LNH	Se+/Le+	−0.580	<0.001
Hex4HexNAc2	Se+/Le+	−0.693	<0.001
LNnDFH	Se+/Le+	0.370	0.036
LNnFP-V	Se–/Le+	−0.480	0.004
LNnFP-V	Se+/Le+	−0.470	<0.001
LNnT	Se+/Le+	−0.604	<0.001
LNT	Se+/Le+	−0.283	0.025
LSTc	Se–/Le+	−1.269	<0.001
LSTc	Se+/Le–	−1.352	0.059
LSTc	Se+/Le+	−1.263	<0.001
MFLNH-III	Se+/Le+	−0.836	<0.001

Only significant and adjusted-for-multiple-testing changes are reported in the table.

*2′FL, 2′-O-Fucosyllactose; 3FL, 3-O-Fucosyllactose; 6′SL, 6′-O-Sialyllactose; DFLNHa, Difucosyllacto-N-hexaose a; LNDFH-I, Lacto-N-difucohexaose-I; LNFP-I,–III, Lacto-N-fucopentaose-I,-III; LNH, Lacto-N-hexaose; Hex, Hexose; HexNAc, N-acetylhexosamine; LNnDFH, Lacto-N-neodifucosylhexaose; LNnFP-V, Lacto-N-neofucopentaose-V; LNnT, Lacto-N-neotetraose; LNT, Lacto-N-tetraose; LSTc, Sialyllacto-N-tetraose c; MFLNH-III, Monofucosyllacto-N-hexaose-III„ Human Milk Oligosaccharide; q-value, level of confidence after correction for multiple HMO testing*.

### Correlations Between HMOs in Our Study Population

**Figure 2:**
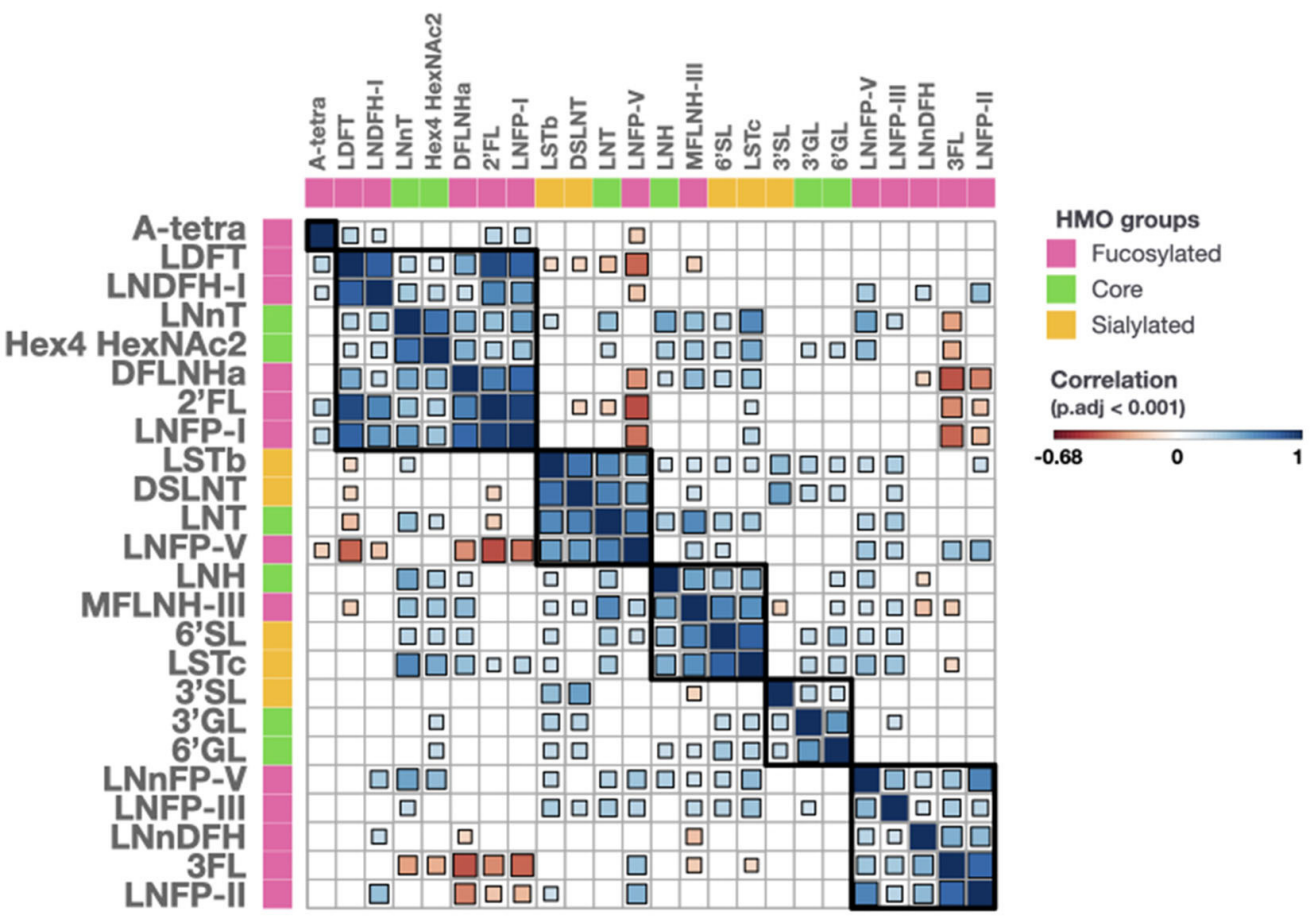
Correlations of all measured HMOs. HMOs are classified into 3 groups, fucosylated (pink), core (green) and sialylated (orange). Correlation values range from −0.68 (dark red) to 1 (dark blue). Size and color of a square are proportional to the correlation coefficient value. Squares are only shown when the adjusted for multiple testing p-value is below 0.001. 2'FL, 2'-O-Fucosyllactose; 3FL, 3-O-Fucosyllactose; 3'GL, 3'-O-Galactosyllactose; 3'SL, 3'-O-Sialyllactose; 6'GL, 6'-O-Galactosyllactose; 6'SL, 6'-O-Sialyllactose; A-tetra, A-tetrasaccharide; DFLNHa, Difucosyllacto-N-hexaose a; DSLNT, Disialyl-lacto-N-tetraose; Hex, Hexose; HexNAc, N-acetylhexosamine; LDFT, Lactodifucotetraose; LNDFH-I, Lacto-N-difucohexaose-I; LNFP-I,-II,-III,-V, Lacto-N-fucopentaose-I,-II,-III,-V; LNH, Lacto-N-hexaose; LNnDFH, Lacto-N-neodifucosylhexaose; LNnFP, Lacto-N-neofucopentaose; LNnFP-V, Lacto-N-neofucopentaose-V; LNnT, Lacto-N-neotetraose; LNT, Lacto-N-tetraose; LSTc, Sialyllacto-N-tetraose c; MFLNH-III, Monofucosyllacto-N-hexaose-III.

We observed significant correlations between several of the HMOs, which could be separated into 5 clusters (Figure 2). Cluster 1 included LDFT, LNDFH-I, DFLNHa, 2′FL, and LNFP-I, all of which contain the same structural feature, α-1,2-fucose, dependent on the activity of FUT2. It also contained LNnT and a hexasaccharide with the composition Hex4HexNAc2, which do not have an obvious connection with the other members of the cluster. Cluster 2 included LSTb, DSLNT, LNT, and LNFP-V. These oligosaccharides are all based on LNT as the core structure elongated with 1 or 2 sialic acids or a fucose. Their concentrations tend to be highest when FUT2 and FUT3 are both inactive as seen in previous studies. Cluster 3 contained LNH, MFLNH-III, 6′SL, and LSTc. MFLNH-III is a fucosylated HMO based on LNH as core. 6'SL and LSTc both contain an α-2,6-linked sialic acid residue. However, the connection between the sialylated structures and the LNH-based structures is not obvious. Cluster 4 contained 3′SL, 3′GL, and 6′GL. All three have the common lactose core with an additional galactose or sialic acid residue. Finally, cluster 5 contained LNnFP-V, LNFP-III, LNnDFH, 3FL, and LNFP-II, all of which are known to contain the structural features α-1,3-fucose or α-1,4-fucose which are dependent on the activity of FUT3. The HMOs in cluster 5 are all present at significantly higher concentrations in milk group 2 and milk group 4 (Supplementary Table 1, *p* < 0.05).

### Representation of the Milk Groups Among Individuals

**Figure 3:**
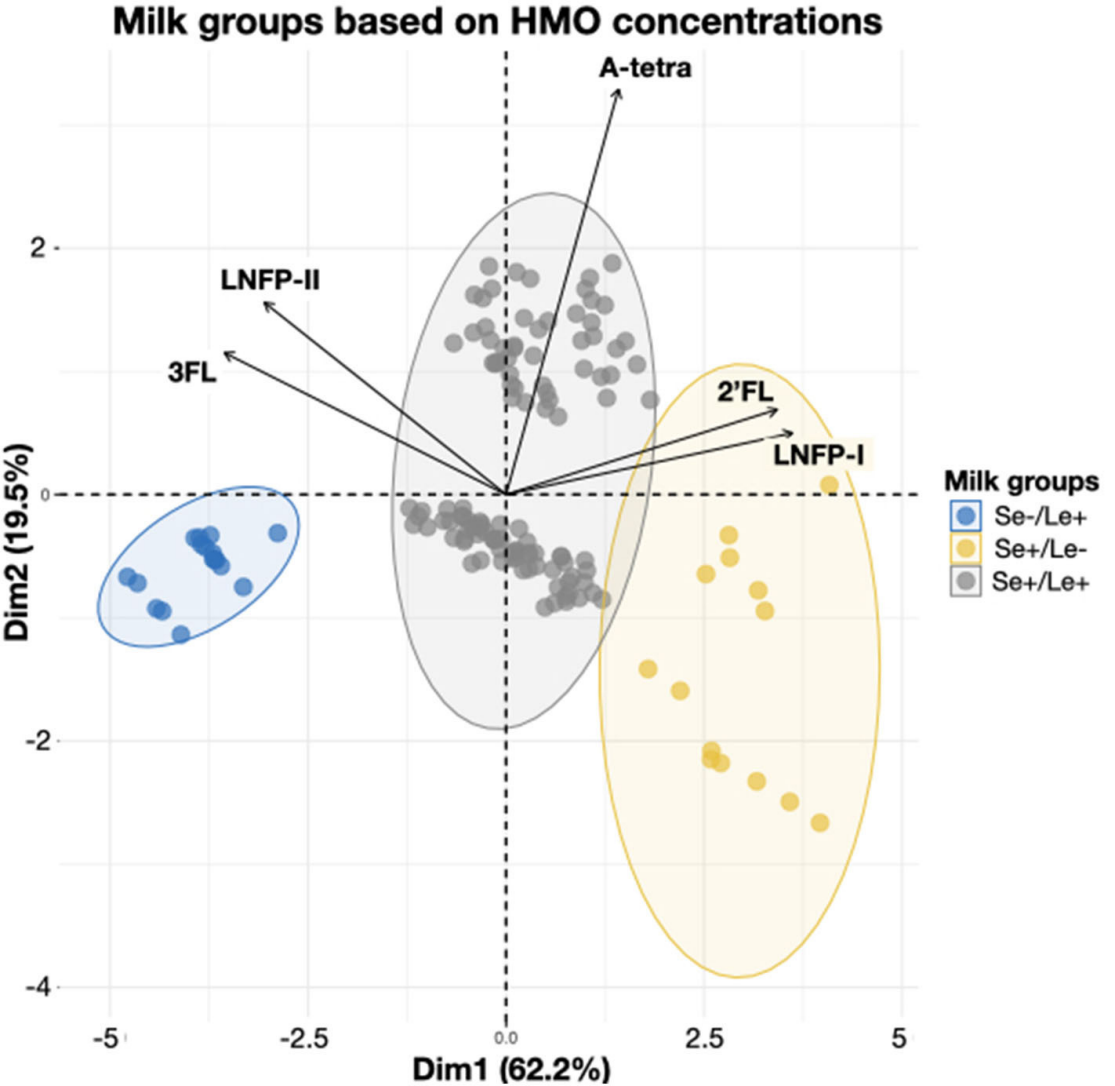
Individual mothers grouped based on 5 HMO concentrations at 3 months. The HMOs concentrations are used to determine the milk group as defined in (22). Secretor-positive groups are even further separated by the presence or absence of A-tetra. Ellipses represent the 95% percentile of confidence.

In order to better understand the relationship between HMOs in lactating mothers at 3 months of lactation, exploratory principal component analysis (PCA) was performed and was found to explain more than 80% of the variance. The first component explained 62.2% of the individual variability and is mainly driven by 3FL and LNFP-II in one direction and by 2′FL and LNFP-I in the other. The first component separates the whole population in 3 distinct groups, which are Se+/Le+, Se+/Le–, and Se–/Le+ (Figure 3). The second component explained 19.5% of the individual variability and is mainly driven by A-tetrasaccharide (A-tetra). Both the Se+/Le+ and the Se+/Le- groups can be separated in the second dimension, while the Se–/Le+ group is unaffected (Figure 3). This makes perfect sense with regards to the A-tetra HMO structure [GalNAcα1-3(Fucα1-2)Galβ1-4Glc], as A-tetra can only be produced by individuals who are Se+ and are blood group A (32).

### Genotypes and Secretor/Lewis Status in the Population Under Study

*FUT2* and *FUT3* exons were sequenced to identify functional single nucleotide polymorphisms (SNPs) affecting HMO profiles in the milk of 156 mothers. Initially, 2,458 SNPs were identified and, after quality check, 20 coding variants were identified in the population (Table 2). The presence of a homozygous genotype for one of the functional variants (i.e., mutation has an effect on enzymatic activity) known to affect Secretor and Lewis status was used to assign mothers as Secretor or non-Secretor and as Lewis positive or Lewis negative. No homozygous mothers were identified for the minor alleles of rs1047781 or rs200157007 in the cohort samples. Therefore, the Secretor status was defined based on SNP rs601338 only. Overall, 134 (88.3%) samples were identified as Secretors and 18 (11.8%) as Non-Secretors.

**Table 2:** The single nucleotide polymorphisms (SNPs) identified in the population after FUT2 and FUT3 exome sequencing.

**SNP**	**CHR**	**chrpos**	**MAF**	**Function**	**Allele**	**AA change**	**Position in protein**	**Affect secretor/Lewis status**
rs3894326	FUT3	5843773	0.07895	Mis.	A>C/T	I>K	356	Yes
rs28362465	FUT3	5844228	0.01316	Syn.	T>C/G	S>S	NA	No
rs3745635	FUT3	5844332	0.02961	Mis.	G/A	G>S	170	Yes
rs778986	FUT3	5844526	0.1908	Mis.	A>G	T>M	105	Yes
rs812936	FUT3	5844638	0.2007	Mis.	G>A/C	W>R/G	68	Yes
rs28362459	FUT3	5844781	0.125	Mis.	A>C/G/T	L>R	20	Yes
rs145362171	FUT3	5844793	0.01645	Mis.	C>G	C>S	16	NA
rs516316	FUT2	48702888	0.3553	IV	G>C	NA	NA	NA
rs516246	FUT2	48702915	0.3553	IV	C>T	NA	NA	NA
rs492602	FUT2	48703160	0.352	Syn.	A>G	A>A	68	No
rs681343	FUT2	48703205	0.3553	SG	C>A/T	Tyr	83	Yes
rs281377	FUT2	48703346	0.4507	Syn.	C>T	N>N	130	No
rs1800022	FUT2	48703368	0.01645	Mis.	C>T	R>C	138	No
rs601338	FUT2	48703417	0.3553	SG	G>A	W>^*^Ter	154	Yes
rs1800027	FUT2	48703469	0.102	Mis.	C>G/T	H>Q	171	Yes
rs602662	FUT2	48703728	0.4046	Mis.	G>A	G>S	258	Yes
rs141630650	FUT2	48703844	0.02303	Syn.	A>A	A>A	296	No
rs485186	FUT2	48703949	0.4046	Syn.	A>G	T>T	331	Yes
rs485073	FUT2	48703998	0.4046	3UV	A>G	NA	NA	NA
rs603985	FUT2	48704000	0.4046	3UV	T>C	NA	NA	NA

*chr, chromosome; chrpos, chromosome position; MAF, Minor Allele Frequency; AA, amino acid; Mis, missense; Syn, synonymous; NA, not analyzed; IV, intron variant; SG, stop gain variant; Ter, termination; FUT2/3, Fucosyltransferase 2/3; 3UV, 3' Untranslated region*.

Lewis status is known to depend on SNPs, rs3745635, rs28362459, rs3894326, and rs812936. In our population, no rs3745635 variants were identified. Therefore, the Lewis status was defined based on SNPs rs28362459, rs3894326, and rs812936. In our study, 9 (5.9%) samples were identified as Lewis negative, and 143 (94.1%) samples were identified as Lewis positive. In 7 samples, rs812936 was found in its minor allele monozygous form C/C and in 2 other samples, rs28362459 was in its minor allele homozygous form G/G. No minor alleles homozygous for rs3894326 were found in the population.

Haplotype analysis for *FUT2* and *FUT3* regions revealed a high linkage disequilibrium LD, *r*^2^ > 0.8 (Supplementary Figure 1).

### HMO Concentrations Are Highly Associated With Genetic Variants

**Figure 4:**
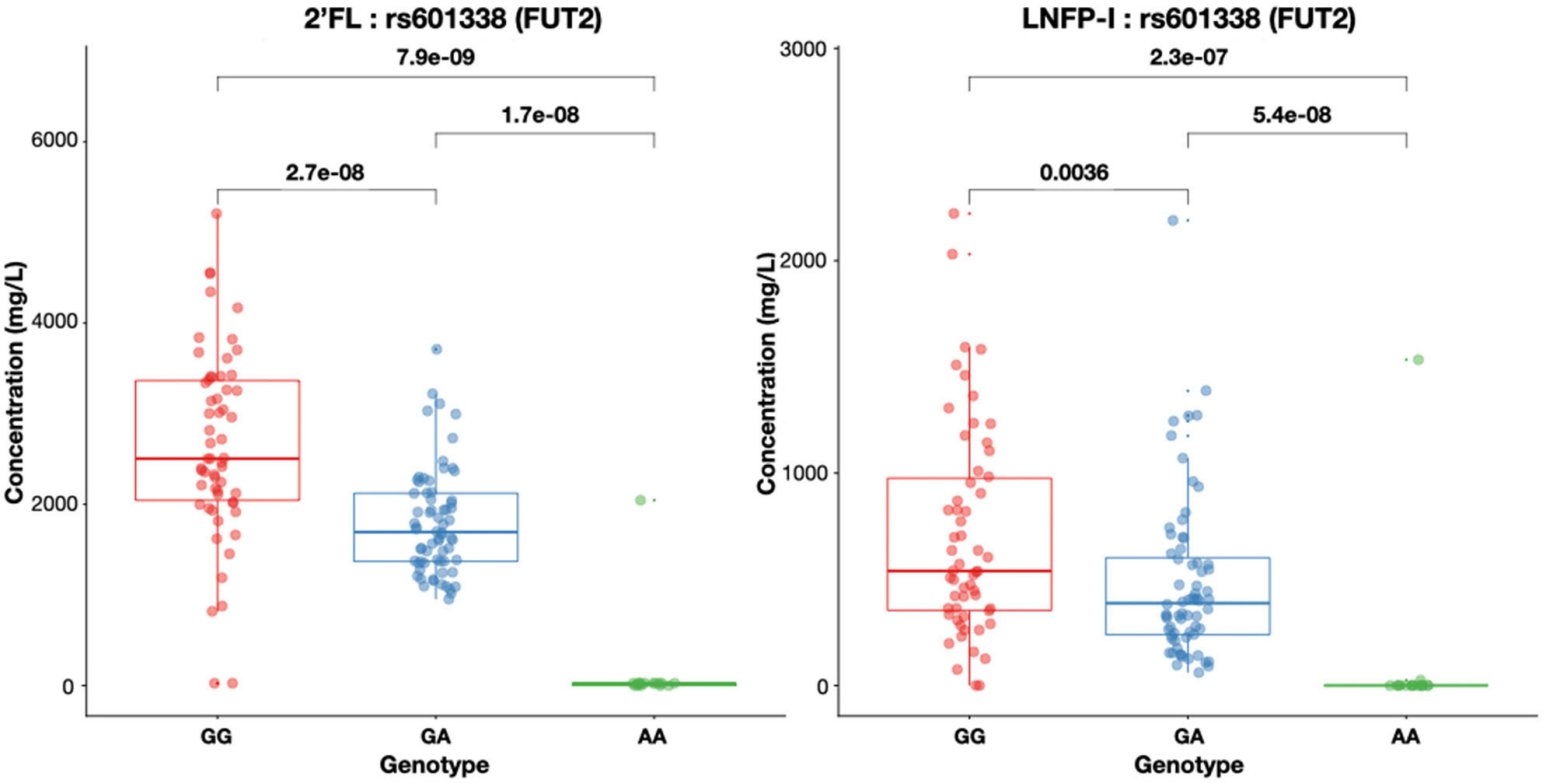
Both 2'FL and LNFP-I concentrations in mg/L in breast milk are significantly different (Mann–Whitney test for non-matched non-parametric data: *P* < 0.0001) depending on rs601338 genotypes. Box plots with median and 25 and 75% interquartiles and 95% CI are shown.

At 3, 6 and 12 months post-partum, we found 2′FL concentrations below LoQ in 13.4, 15.9, and 10.7% of the mothers, respectively. LNFP-II concentrations at 3, 6, and 12 months were below LoQ in 10.8, 12.6, and 14.7% of the mothers, respectively. Secretor and Lewis status based on HMO concentrations did not differ among the 3 time-points for the same individual. We sought to correlate HMO concentrations with *FUT2* and *FUT3* non-functional variants. Concentrations of individual HMOs dependent on Secretor or Lewis status were associated with *FUT2* and *FUT3* genotypes of the individual, respectively. Rs601338 was significantly associated with the concentrations of both 2′FL and LNFP-I in breast milk (Figure 4). Individuals with the wild type G/G genotype have high concentrations of 2′FL in their milk (Figure 4). Indeed, for most samples identified as Non-Secretors by the presence of rs601338 variations, 2′FL concentrations were below LoQ <20 mg/L. Two samples genetically identified as Secretors (G/G) also had 2′FL concentrations below LoQ. A single sample was indentifed as Non-Secretor (A/A) and 2′FL concentration was higher than LoQ. For both results, there was no match between the genetic and the HMO analysis. We lowered the threshold of MAF to 1% to identify rare and potentially missense variants to explain these results, but we did not identify any novel FUT2 SNPs in this population located at exon regions (data not shown). Similar results as for 2′FL were observed for LNFP-I, another major FUT2-dependent HMO (Figure 4).

**Figure 5:**
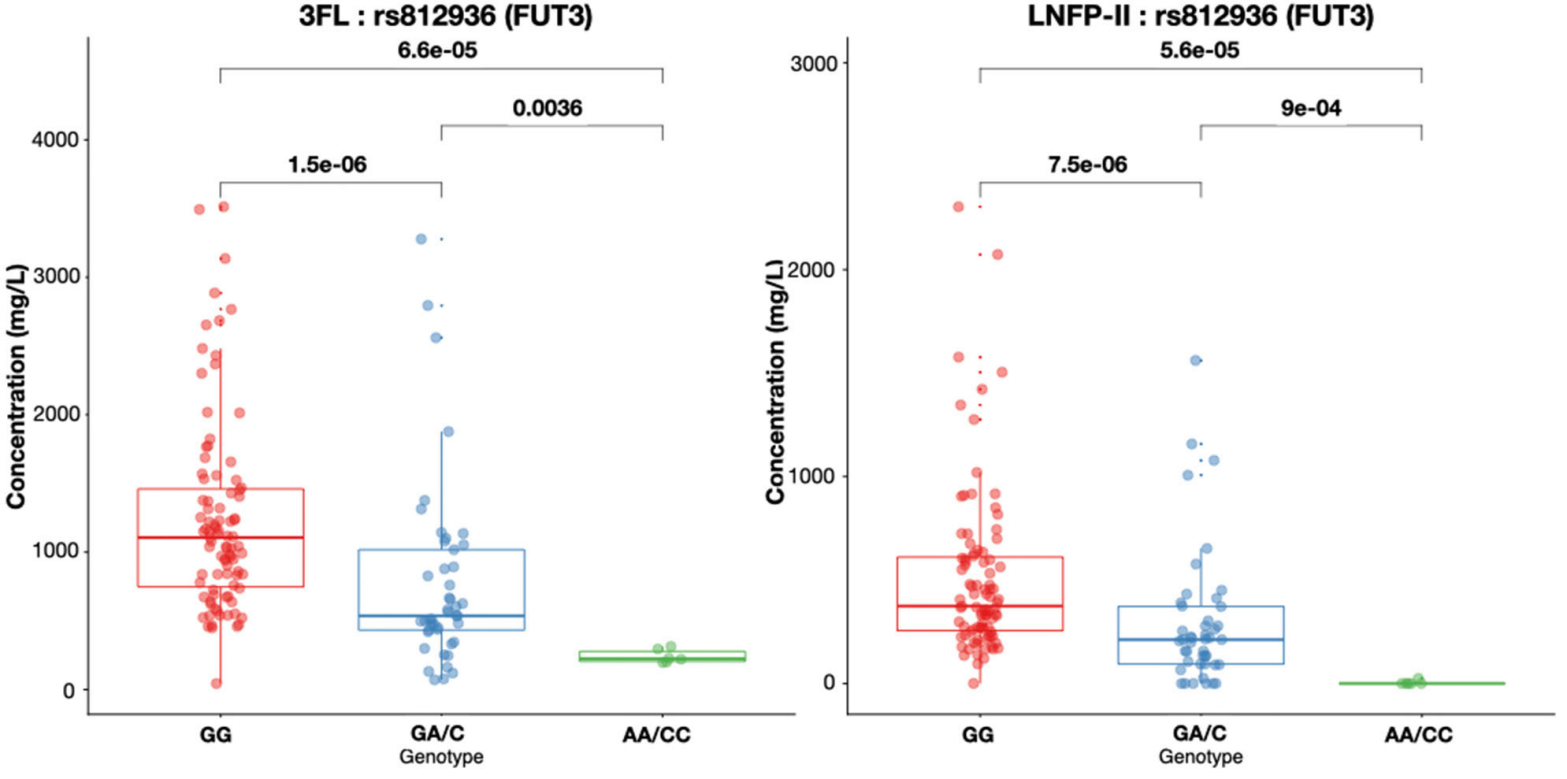
3FL and LNFP-II concentrations in mg/L in breast milk according to rs812936 genotypes. Box plots with median and 25 and 75% interquartiles and 95% CI are shown.

Rs812936, the main genetic determinant of Lewis status, was associated with LNFPII concentration. Indviduals with G/G genotype had higher concentrations of LNFPII in their milk (Figure 5). For the 8 samples identified as Lewis negative based on rs28362459 and rs812936 SNPs, LNFP-II was not detected in the milk. However, in the population, 8 samples were identified as Lewis positive based on rs28362459, rs3894326, rs812936 (wild type allele homozygotes or heterozygotes), but had no detectable LNFP-II in the respective milk samples. For 6 of them, two or more missense *FUT3* variants were in their heterozygote form with rs812936 and rs778986 always in their heterozygote form T/C and C/T, respectively. From our data, we could not provide an explanation for the remaining 2 samples.

### Genetic Predictors of Milk Groups

**Figure 6:**
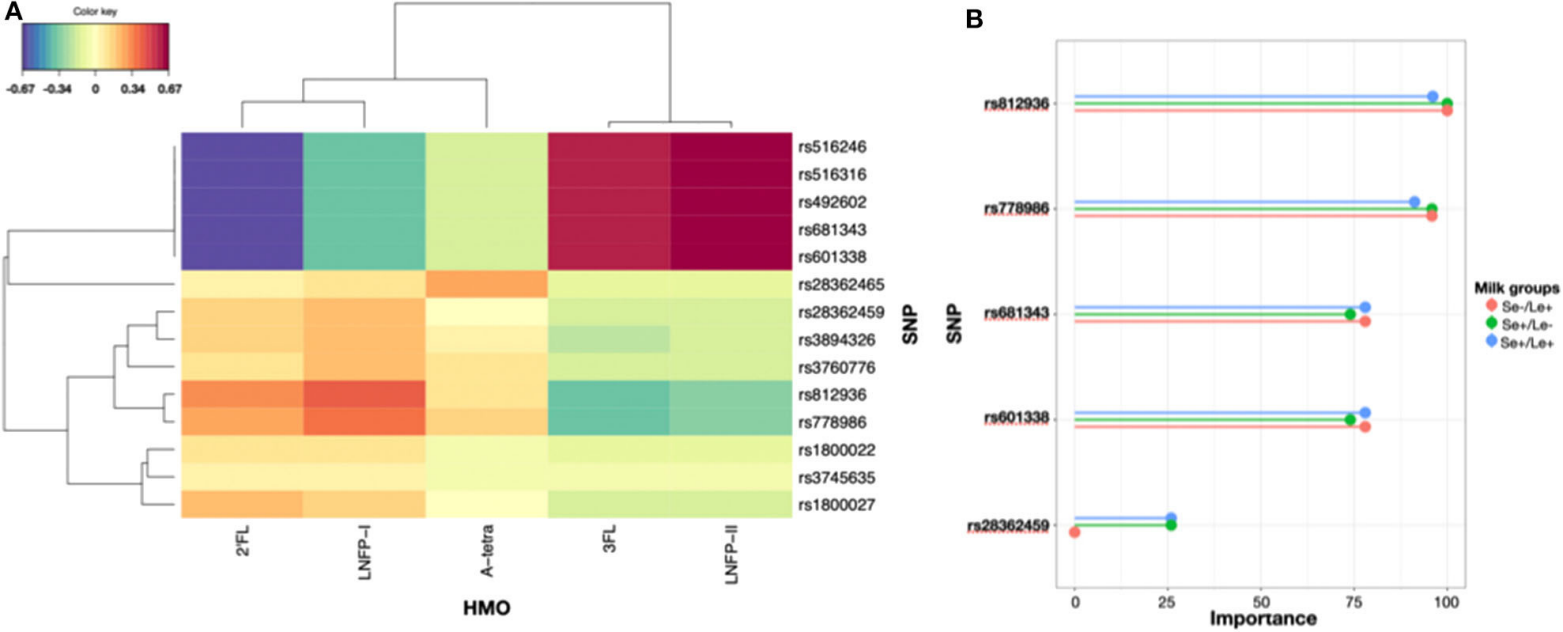
**(A)** sPLS-selected genetic polymorphisms and their associations with HMO levels expressed as a distance between genotypes and HMO levels in 142 individuals. **(B)** Importance of genetic polymorphisms in predicting the milk group of mothers with a MultiLayer Perceptron model.

There were genetic polymorphisms in the *FUT2* and *FUT3* genes that were associated with the concentrations of major HMOs. We showed that rs516246, rs516316, rs492602, rs681343, and rs601338 were associated with low concentrations of 2′FL, and LNFP-I while being associated with high concentrations of 3FL and LNFP-II. On the other hand and to a lesser extent, rs28362459, rs3894326, rs3760776, rs812936, rs778986, rs1800022, rs3745635, and rs1800027 were associated with high concentrations of 2′FL and LNFP-I and low concentrations of 3FL and LNFP-II. Interestingly, rs128362465 had a tendency to be associated with A-tetra concentrations (Figure 6A).

In order to predict the milk group of future mothers, we fitted a MLP model based on genetic polymorphisms and showed that rs812936, rs778986, rs681343, rs601338, rs28362459 were top-predictors of the milk groups with an average accuracy of 0.978 (Figure 6B, Supplementary Table 3).

### A Genetic Score to Predict 2′FL Concentrations in Milk for Future Secretor Mothers

**Figure 7:**
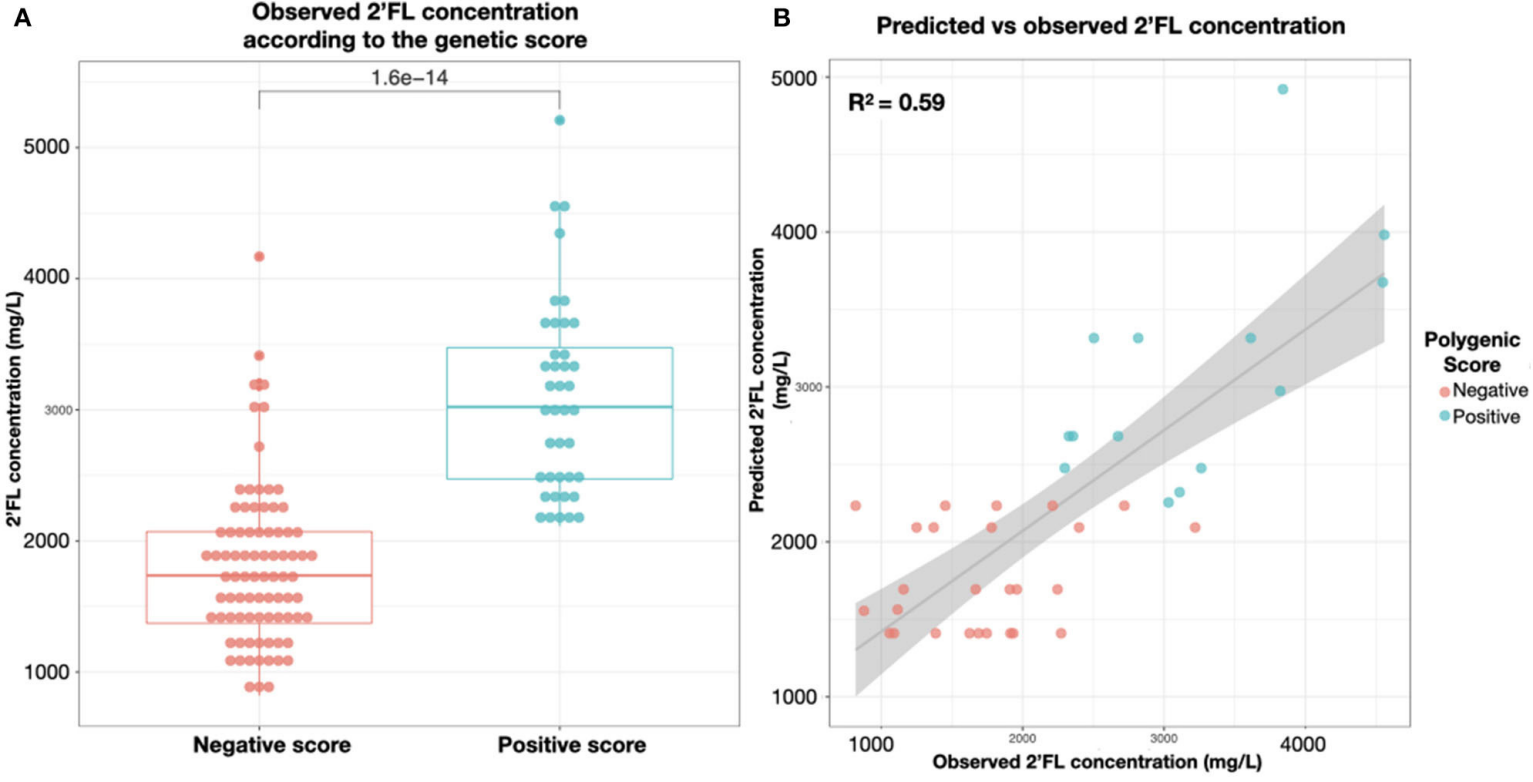
**(A)** 2′FL concentrations in milk (mg/L) of the 124 secretor mothers by genetic score categorization in high secretors positive score and moderate secretors zero or negative score. **(B)** In an independent test dataset, correlation between genetic score predicted and observed concentrations of 2'FL (mg/L) secreted in the milk of the individual mothers.

We developed a genetic score to demonstrate the additive impact of genetic polymorphisms of *FUT2* and *FUT3* genes on the concentrations of 2′FL, the most abundant HMO in Secretor milk. Interestingly, we showed that the Secretor population could be divided into two sub-populations with moderate and high levels of 2′FL, and that we could predict fairly well the 2′FL concentrations in a mother's milk based on her genetic score (Adjusted-*R*^2^ = 0.58, *p* < 6.6.10^−9^). A zero or negative score predicted a moderate 2′FL concentration, while a positive score predicted a high amount of 2′FL in her milk (Table 3 and Figures 7A,B). Though 2′FL concentrations were mainly associated with rs601338 polymorphism and heterozygous mothers were predominatly represented in the moderate level group, our polygenic score significantly outperformed predictions based on rs601338 alone (*p* < 0.001).

**Table 3:** Summary table for the SNPs included in the genetic score.

**SNP**	**Coefficient**	**Standard error**	***P*-value**
rs601338	−0.56304	0.06936	<0.001
rs28362459	0.39504	0.06534	<0.001
rs778986	0.18314	0.04536	<0.001
rs1800022	−0.36211	0.14316	0.013
rs281377	0.10313	0.05702	0.074

## Discussion

Our study provides for the first time detailed and systematic insight on the link between the breast milk HMO concentrations and maternal *FUT2* and *FUT3* genetic variants. We focused our analysis on exonic genetic variants to identify the ones with a functional role on FUT2 and FUT3 enzyme activities. In this population, we identified 1 known missense SNP on *FUT2*, rs601338, responsible for the non-Secretor phenotype in milk and 3 known missense SNPs, rs28362459, rs3894326, and rs812936 on *FUT3*, responsible for the Lewis negative phenotype in milk.

SNP rs601338, the predominant *FUT2* variant has a minor allele frequency (MAF) in European and African populations ranging from 30 to 57% (1,000 Genomes Project, https://www.internationalgenome.org/1000-genomes-browsers). This SNP has a very low frequency 0–1% in South East Asian populations, whereas in Indian populations, the average MAF is 25% (1,000 Genomes Project). Generally, in our population the samples genetically identified as non-Secretors had an A/A genotype and, as expected, measured 2′FL and LNFP-I concentrations below LoQ. Two samples genetically identified as Secretors G/G also had 2′FL and LNFP I below LoQ, actually close to their LoD (limit of detection) of 3.9 and 2 mg/L for 2′FL and LNFP-I, respectively (29). We investigated our dataset by looking for novel rare *FUT2* non-coding variants, but we did not identify any that could explain these results. It is possible that other *FUT2* SNPs outside the exonic regions, perhaps with a regulatory function could define the enzyme expression. To our knowledge, the FUT2 enzyme is the only known fucosyltransferase that generates α-1,2-fucosylated HMOs. Hypothetically, another unknown fucosyltransferase may be active in the mammary gland explaining our observation of 2 samples identified genetically as Secretors, but expressing only very small amount of 2′FL. Another possibly more likely explanation is the presence of mutations in regulatory elements for *FUT2* that we did not capture in our analysis. A future approach would be to perform an expression quantitative trait loci (eQTL) to identify *cis*- or *trans*-eQTLs that may affect the expression of the *FUT2* gene and then verify this by testing their correlation with 2′FL concentrations. Furthermore, a genome wide approach to find genetic variants associated with 2′FL concentrations could provide further insight into the unknown variation controlling the expression of the enzyme.

We used the same approach to identify the link between *FUT3* exonic variants and α1,3-4-fucosylated HMO, like LNFP-II and 3FL. In our population, most of the Lewis negative mothers were homozygotes for one of the three functional *FUT3* SNPs rs28362459, rs3894326, rs812936. In contrast to Secretor status, the Lewis status seems to be defined by a higher number of SNPs which are also less well characterized than *FUT2* (33, 34). SNP rs28362459 occurs more frequently in South East Asian, Indian and African populations (MAF = 25 to 35%) compared to European populations (MAF = 10%). SNP rs3894326 is more common in Asian populations (MAF = 15%) compared to European and African (MAF > 7%). Finally, SNP rs812936 is not frequent in South East Asian populations (MAF = 3%) but ranges from 10 to 20% in all other populations included in the 1000 Genomes Project 1000 Genomes Project, (https://www.internationalgenome.org/1000-genomes-browsers). In our cohort, we identified 8 mothers as Lewis positive based on the three SNPs rs28362459, rs3894326 and rs812936, yet no LNFP-II was detected in their milk. Interestingly, 6 of them were heterozygotes for both rs812936 and rs778986 SNPs, indicating that the phenotype may be a result of compound heterozygosity (35). However, we could not analyze phased genotypes in our dataset to explore this possibilty, due to high linkage disequilibrium in the region. Future studies need to explore whether *FUT2* and *FUT3* genetic variants are subjected to compound heterozygosity explaining the missing production of specific HMOs despite an apparent functional genotype.

In this population of European mothers, those with the heterozygous forms of the Secretor and Lewis status-defining SNPs appear to have intermediate levels of the dependent HMOs like 2′FL, LNFP-I, LNFP-II, and 3FL compared to higher levels in the major allele homozygous functional forms. This may have several implications for research on HMO biology and clinical relevance and may explain part of the large variability observed for many HMOs in breast milk. In an attempt to further explain variability of HMOs by combined *FUT2* and *FUT3* variations, we constructed a genetic score to predict the concentrations of 2′FL. Indeed, we observed that 5 SNPs located in both genes were able to predict 2′FL concentrations in breast milk. The finding did not seem to be solely dependent on presence of *FUT2* non-functional SNP. Instead, the combination of both *FUT2* and *FUT3* variation were needed to explain the variability in 2′FL levels, confirming the hypothesis that final concentrations of specific individual HMOs are influenced by the balance between FUT2 and FUT3 expression (1), as well as donor and acceptor substrate availability for the respective enzymes.

Similarly, our results showed that 5 *FUT2* and *FUT3* SNPs are sufficient to predict the milk groups in the population. Our population was homogenous with European ancestry and it would be important that these relationships between *FUT2* and *FUT3* genetic variants and HMOs are confirmed in admixed or populations with different ancestry, e.g., Asian (17).

We also report here how HMOs cluster and change in concentration over the course of lactation until 12 months of age. Albeit at 12 months of age our sample size was relatively small (*N* = 28), these data still provide a valuable complement to previously published studies reporting concentrations of HMOs beyond 6 months of lactation that generally had even lower sample sizes (36, 37). Overall concentrations for most HMOs decrease over time of lactation with some changes being statistically significant like for 6′SL, LST-c, and MFLNH-III. On the other hand, concentrations of 3FL and LNnDFH increase from 6 to 12 months. Certain HMOs are highly correlated with each other like the FUT2-dependent-HMOs, which are inversely correlated with FUT3-dependent and some sialylated HMOs. Overall the results reflect the dependence on specific fucosyltransferases and the substrate competition for these enzymes reported before (1). We observed several clusters showing some expected relations between HMOs, like a FUT2 or FUT3 dependence, but the clusters also showed some unexpected relations. For example, LNnT clustered with the FUT2 dependent HMOs 2′FL and LNFP-I and 3′SL clustered with the galactosyllactoses 3′GL and 6′GL. Very few studies have analyzed HMOs up to 12 months of lactation (37). Gridneva et al. (37) reported that although total measured HMOs slightly decreased over time, this is not statistically significant, a result similar to ours. Individual HMOs or groups of HMOs, however, may have a more dynamic profile over time, but this was not reported in that study. Generally, for HMOs like 3FL, the increase in concentration over time may reflect a role relevant to later developmental stages. Yet, today no such associations were reported in the literature as far as we know.

We found that genetic characterization by milk groups was a strong factor explaining the HMO distribution and that 5 individual HMOs 2′FL, LNFP-I, A-tetra, 3FL, and LNFP-II were sufficient to characterize these clusters. Within the clusters, smaller subgroups were visible, mainly driven by A-tetra. This may explain a recent report showing that within Secretors smaller subgroups are present (38). Actually, A-tetra appears only in milk of Secretor mothers, who are also of the blood group A type meaning they have a functional N-acetylgalactosamine transferase that can add GalNAc to H-type glycans like 2′FL for example (32).

Clearly, HMO concentrations are strongly determined by genetic factors, namely SNPs on *FUT2* and *FUT3* and their combinations. Consequently, these factors should be considered when exploring HMO compositional variation in relation to other maternal factors and diet. Yet, unidentified rare variation and organization of genomic regions need also to be further explored and possibly accounted for. In addition, further large studies are needed to identify currently unknown regulatory variations that may impact the function of these fucosyltransferases or other enzymes involved in the production of HMOs. Such additional factors may be able to better explain the temporal dynamic changes and the large inter-individual variability seen in several observational studies. Ultimately, information explaining HMO variability is important to better understand and interpret HMO effects observed in relation to growth and health measures in breastfed infants at different developmental stages, as some like the maternal genetic factors are also linked to the infants genetic makeup.

## Data Availability Statement

The datasets presented in this study can be found in online repositories. The names of the repository/repositories and accession number(s) can be found at: https://www.ncbi.nlm.nih.gov/bioproject/PRJNA643141.

## Ethics Statement

The studies involving human participants were reviewed and approved by University of Leipzig Faculty of Medicine. Written informed consent to participate in this study was provided by the participants' legal guardian/next of kin.

## Author Contributions

GL conducted statistical analysis and drafted the manuscript. MS conducted statistical analysis and reviewed the manuscript. AC conducted laboratory analysis and reviewed the manuscript. JM designed and conducted laboratory analysis and reviewed the manuscript. MV and WK designed the study and reviewed the manuscript. TK analyzed data and reviewed the manuscript. SA designed the study and drafted the manuscript. NS conceived and designed the analysis and drafted the manuscript. AB concieved and designed the study and drafted the manuscript. All authors contributed to the article and approved the submitted version.

## Conflict of Interest

GL, MS, AC, JM, SA, NS, and AB were employees of Societe des Produits Nestl. SA during the time of the study. The remaining authors declare that the research was conducted in the absence of any commercial or financial relationships that could be construed as a potential conflict of interest

## Abbreviations

2′FL: 2′-O-Fucosyllactose
3FL: 3-O-Fucosyllactose
3′GL: 3′-Galactosyllactose
3'SL: 3′-O-Sialyllactose
6′GL: 6′-Galactosyllactose
6'SL: 6′-O-Sialyllactose
AA: Amino acid
A-tetra: A-tetrasaccharide
Chr: Chromosome
Chrpos: Chromosome position
DFLNHa: Difucosyl-lacto-N-hexaose a
DSLNT: Di-Sialyl-lacto-N-tetraose
FUT2: Fucosyltransferase 2
FUT3: Fucosyltransferase 3
GLM: Generalized Linear Model
Hex: Hexose
HexNAc: N-acetylhexosamine
HMO: Human milk oligosaccharides
IV: Intron variant
LD: Linkage disequilibrium
LDFT: Lactodifucotetraose
Le: Lewis
LNDFH-I: Lacto-N-di-fucohexaose I
LNFP-I,-II,-III,-V: Lacto-N-fucopentaose-I,-II,-III,-V
LNH-a,-b: Lacto-N-hexaose-a,-b
LNnDFH: Lacto-N-neodifucosylhexaose
LNnFP: Lacto-N-neofucopentaose
LNnFP-V: Lacto-N-neofucopentaose V
LNnT: Lacto-N-neo-tetraose
LNT: Lacto-N-tetraose
LSTc: Sialyl-lacto-N-tetraose c
LoQ: Level of Quantification
MAF: Minor allele frequency
MFLNH-III: Monofucosyl-lacto-N-hexaose III
Mis: Missense
NS: Not analyzed
sPLS: Sparse Partial Least Square
Se: Secretor
SG: Stop gain
SNP: Single nucleotide polymorphism
Syn: Synonymous.
