# Supplementary material for: Time of Lactation and Maternal Fucosyltransferase Genetic Polymorphisms Determine the Variability in Human Milk Oligosaccharides
Source: Front Nutr. 2020 Oct 29;7:574459. doi: 10.3389/fnut.2020.574459 (PMC7658960; doi:10.3389/fnut.2020.574459)
Supplement: Supplementary file 1 [file Table_3.DOCX]

**Supplementary material**

Supplemental Figures

**Sup figure 1**. Heatmap of FUT2 and FUT3 gene exonic regions showing correlation expressed as R*^2^* based on EU reference population between polymorphisms detected in our population. Each of the two regions show a high level of linkage disequilibrium.

*(https://ldlink.nci.nih.gov)*

**Sup figure 2**. Evaluation of the prediction models performance based on accuracy and recall rate.

**Supplemental Tables**

**Supp table I.** Concentrations of HMO, per time-point and in different milk groups expressed as median and high/low (mg/ml).

| **HMO** | **Milk group** | **3 months (mg/L)** | | | | **6 months (mg/L)** | | | | **12 months (mg/L)** | | | |
| --- | --- | --- | --- | --- | --- | --- | --- | --- | --- | --- | --- | --- | --- |
|  |  | **Min** | **Median** | **Mean** | **Max** | **Min** | **Median** | **Mean** | **Max** | **Min** | **Median** | **Mean** | **Max** |
| 2'FL | Se-/Le+ | 0 | 5 | 6 | 17 | 0 | 2 | 3 | 10 | 0 | 2 | 2 | 4 |
| 2'FL | Se+/Le- | 1391 | 3368 | 3322 | 5209 | 1253 | 3232 | 3054 | 4394 | 1737 | 3807 | 3465 | 4850 |
| 2'FL | Se+/Le+ | 819 | 1999 | 2120 | 4547 | 736 | 1688 | 1798 | 3876 | 694 | 1430 | 1561 | 2950 |
| 3FL | Se-/Le+ | 1657 | 2653 | 2634 | 3647 | 1592 | 3047 | 3257 | 6032 | 2481 | 3429 | 3429 | 4377 |
| 3FL | Se+/Le- | 0 | 192 | 172 | 314 | 8 | 235 | 224 | 455 | 85 | 404 | 306 | 429 |
| 3FL | Se+/Le+ | 299 | 869 | 914 | 2018 | 393 | 1123 | 1257 | 3384 | 780 | 1294 | 1377 | 2343 |
| 3'GL | Se-/Le+ | 1 | 3 | 4 | 14 | 1 | 2 | 2 | 6 | 2 | 2 | 2 | 2 |
| 3'GL | Se+/Le- | 1 | 3 | 3 | 4 | 1 | 2 | 3 | 4 | 3 | 4 | 4 | 5 |
| 3'GL | Se+/Le+ | 1 | 3 | 4 | 38 | 0 | 2 | 2 | 14 | 1 | 3 | 3 | 5 |
| 3'SL | Se-/Le+ | 103 | 145 | 153 | 282 | 108 | 155 | 176 | 365 | 136 | 212 | 212 | 287 |
| 3'SL | Se+/Le- | 89 | 121 | 133 | 191 | 90 | 137 | 157 | 322 | 127 | 155 | 262 | 503 |
| 3'SL | Se+/Le+ | 64 | 124 | 130 | 348 | 31 | 120 | 129 | 428 | 87 | 200 | 249 | 929 |
| 6'GL | Se-/Le+ | 7 | 15 | 18 | 57 | 3 | 9 | 12 | 52 | 9 | 9 | 9 | 9 |
| 6'GL | Se+/Le- | 7 | 12 | 15 | 42 | 6 | 9 | 12 | 27 | 6 | 9 | 9 | 11 |
| 6'GL | Se+/Le+ | 4 | 13 | 16 | 258 | 0 | 8 | 9 | 26 | 6 | 11 | 11 | 18 |
| 6'SL | Se-/Le+ | 38 | 125 | 162 | 481 | 18 | 44 | 51 | 92 | 17 | 24 | 24 | 31 |
| 6'SL | Se+/Le- | 62 | 134 | 136 | 244 | 11 | 35 | 54 | 114 | 16 | 21 | 37 | 75 |
| 6'SL | Se+/Le+ | 37 | 116 | 127 | 275 | 8 | 40 | 47 | 213 | 5 | 14 | 26 | 139 |
| A-tetra | Se-/Le+ | 0 | 0 | 0 | 0 | 0 | 0 | 1 | 11 | 0 | 0 | 0 | 0 |
| A-tetra | Se+/Le- | 0 | 0 | 64 | 726 | 0 | 0 | 53 | 486 | 0 | 51 | 165 | 445 |
| A-tetra | Se+/Le+ | 0 | 0 | 47 | 705 | 0 | 6 | 43 | 289 | 0 | 1 | 20 | 117 |
| DFLNHa | Se-/Le+ | 0 | 3 | 3 | 15 | 0 | 5 | 5 | 22 | 0 | 3 | 3 | 6 |
| DFLNHa | Se+/Le- | 33 | 231 | 242 | 500 | 14 | 126 | 121 | 276 | 83 | 94 | 106 | 140 |
| DFLNHa | Se+/Le+ | 5 | 52 | 65 | 259 | 0 | 20 | 27 | 122 | 3 | 13 | 16 | 55 |
| DSLNT | Se-/Le+ | 97 | 210 | 222 | 559 | 71 | 174 | 181 | 345 | 145 | 197 | 197 | 249 |
| DSLNT | Se+/Le- | 85 | 127 | 154 | 264 | 67 | 101 | 134 | 301 | 143 | 218 | 212 | 274 |
| DSLNT | Se+/Le+ | 41 | 155 | 164 | 546 | 34 | 117 | 124 | 330 | 76 | 155 | 166 | 351 |
| LDFT | Se-/Le+ | 0 | 0 | 1 | 8 | 0 | 1 | 4 | 37 | 0 | 1 | 1 | 2 |
| LDFT | Se+/Le- | 8 | 108 | 104 | 173 | 14 | 111 | 130 | 290 | 112 | 129 | 271 | 573 |
| LDFT | Se+/Le+ | 148 | 328 | 379 | 1506 | 161 | 332 | 383 | 1612 | 145 | 382 | 509 | 1209 |
| LNDFH-I | Se-/Le+ | 0 | 0 | 1 | 3 | 0 | 0 | 9 | 76 | 0 | 2 | 2 | 3 |
| LNDFH-I | Se+/Le- | 0 | 0 | 9 | 67 | 0 | 0 | 5 | 54 | 0 | 2 | 3 | 8 |
| LNDFH-I | Se+/Le+ | 57 | 917 | 970 | 2773 | 239 | 645 | 695 | 1452 | 302 | 729 | 765 | 1476 |
| LNFP-I | Se-/Le+ | 0 | 0 | 0 | 1 | 0 | 0 | 2 | 31 | 0 | 0 | 0 | 0 |
| LNFP-I | Se+/Le- | 621 | 1121 | 1221 | 2222 | 355 | 881 | 919 | 1896 | 1413 | 1418 | 1418 | 1422 |
| LNFP-I | Se+/Le+ | 61 | 412 | 533 | 2031 | 0 | 243 | 338 | 1520 | 39 | 200 | 254 | 671 |
| LNFP-II | Se-/Le+ | 525 | 1158 | 1268 | 2304 | 335 | 799 | 820 | 1513 | 647 | 720 | 720 | 792 |
| LNFP-II | Se+/Le- | 0 | 0 | 1 | 10 | 0 | 0 | 3 | 15 | 0 | 0 | 0 | 0 |
| LNFP-II | Se+/Le+ | 66 | 278 | 335 | 917 | 52 | 249 | 272 | 662 | 96 | 284 | 286 | 720 |
| LNFP-III | Se-/Le+ | 272 | 456 | 481 | 835 | 234 | 343 | 378 | 777 | 181 | 239 | 239 | 296 |
| LNFP-III | Se+/Le- | 27 | 310 | 329 | 578 | 29 | 296 | 285 | 496 | 166 | 264 | 295 | 455 |
| LNFP-III | Se+/Le+ | 189 | 386 | 407 | 886 | 127 | 314 | 324 | 575 | 124 | 261 | 253 | 433 |
| LNFP-V | Se-/Le+ | 67 | 158 | 156 | 270 | 51 | 104 | 112 | 198 | 91 | 95 | 95 | 98 |
| LNFP-V | Se+/Le- | 4 | 26 | 28 | 60 | 8 | 24 | 27 | 57 | 30 | 32 | 36 | 46 |
| LNFP-V | Se+/Le+ | 11 | 40 | 45 | 117 | 4 | 34 | 37 | 92 | 10 | 35 | 35 | 72 |
| LNH-a | Se-/Le+ | 0 | 24 | 25 | 48 | 0 | 17 | 18 | 41 | 0 | 11 | 11 | 22 |
| LNH-a | Se+/Le- | 6 | 23 | 25 | 55 | 6 | 11 | 11 | 21 | 6 | 7 | 7 | 8 |
| LNH-a | Se+/Le+ | 0 | 27 | 30 | 98 | 2 | 11 | 15 | 48 | 0 | 11 | 11 | 27 |
| LNH-b | Se-/Le+ | 0 | 8 | 10 | 30 | 0 | 3 | 7 | 33 | 3 | 11 | 11 | 19 |
| LNH-b | Se+/Le- | 3 | 12 | 25 | 147 | 0 | 6 | 16 | 101 | 0 | 12 | 10 | 17 |
| LNH-b | Se+/Le+ | 1 | 20 | 24 | 89 | 0 | 8 | 13 | 84 | 0 | 8 | 10 | 46 |
| LNnDFH | Se-/Le+ | 0 | 0 | 63 | 183 | 56 | 124 | 191 | 642 | 83 | 272 | 272 | 461 |
| LNnDFH | Se+/Le- | 0 | 15 | 17 | 42 | 0 | 10 | 9 | 30 | 0 | 6 | 13 | 32 |
| LNnDFH | Se+/Le+ | 3 | 27 | 36 | 164 | 6 | 47 | 51 | 150 | 21 | 37 | 48 | 115 |
| LNnFP-V | Se-/Le+ | 5 | 20 | 19 | 41 | 3 | 12 | 12 | 23 | 5 | 8 | 8 | 10 |
| LNnFP-V | Se+/Le- | 1 | 2 | 3 | 8 | 0 | 2 | 2 | 5 | 0 | 4 | 5 | 11 |
| LNnFP-V | Se+/Le+ | 4 | 15 | 17 | 64 | 3 | 9 | 12 | 48 | 2 | 5 | 5 | 13 |
| LNnT | Se-/Le+ | 15 | 51 | 59 | 138 | 10 | 30 | 36 | 82 | 9 | 23 | 23 | 36 |
| LNnT | Se+/Le- | 32 | 79 | 118 | 312 | 18 | 54 | 85 | 270 | 24 | 55 | 47 | 63 |
| LNnT | Se+/Le+ | 36 | 128 | 151 | 471 | 13 | 82 | 100 | 354 | 11 | 37 | 47 | 98 |
| LNT | Se-/Le+ | 347 | 743 | 773 | 1284 | 136 | 442 | 547 | 1252 | 259 | 416 | 416 | 572 |
| LNT | Se+/Le- | 194 | 442 | 510 | 1274 | 120 | 343 | 431 | 1170 | 269 | 377 | 595 | 1138 |
| LNT | Se+/Le+ | 161 | 555 | 613 | 1694 | 54 | 388 | 449 | 1529 | 133 | 346 | 354 | 593 |
| LSTc | Se-/Le+ | 0 | 31 | 43 | 114 | 3 | 8 | 9 | 28 | 0 | 1 | 1 | 2 |
| LSTc | Se+/Le- | 12 | 58 | 52 | 92 | 3 | 13 | 14 | 28 | 0 | 5 | 5 | 10 |
| LSTc | Se+/Le+ | 12 | 51 | 76 | 1331 | 0 | 13 | 17 | 84 | 0 | 3 | 6 | 26 |
| MFLNH-III | Se-/Le+ | 75 | 151 | 180 | 345 | 8 | 67 | 74 | 200 | 18 | 22 | 22 | 26 |
| MFLNH-III | Se+/Le- | 75 | 173 | 190 | 325 | 21 | 77 | 86 | 161 | 31 | 36 | 69 | 141 |
| MFLNH-III | Se+/Le+ | 29 | 129 | 133 | 374 | 4 | 59 | 67 | 198 | 6 | 22 | 38 | 226 |

**Supp table II.** Changes of HMO during lactation by milk group. The results were corrected for multiple testing (q-value).

| **HMO** | **Milk group** | **coefficient** | **p-value** | **q-value** |
| --- | --- | --- | --- | --- |
| 2'FL | Se-/Le+ | -6.93E-01 | 1.95E-01 | 1.00E+00 |
| 2'FL | Se+/Le- | 1.10E-01 | 5.19E-01 | 1.00E+00 |
| 2'FL | Se+/Le+ | -1.67E-01 | 1.01E-03 | 5.13E-02 |
| 3FL | Se-/Le+ | 1.38E-01 | 1.73E-01 | 1.00E+00 |
| 3FL | Se+/Le- | 2.48E-01 | 3.57E-01 | 1.00E+00 |
| 3FL | Se+/Le+ | 2.04E-01 | 1.86E-03 | 8.72E-02 |
| 3'GL | Se-/Le+ | -2.88E-01 | 1.08E-01 | 1.00E+00 |
| 3'GL | Se+/Le- | 0.00E+00 | 1.00E+00 | 1.00E+00 |
| 3'GL | Se+/Le+ | -1.44E-01 | 1.65E-01 | 1.00E+00 |
| 3'SL | Se-/Le+ | 6.62E-02 | 6.17E-01 | 1.00E+00 |
| 3'SL | Se+/Le- | 1.23E-01 | 3.60E-01 | 1.00E+00 |
| 3'SL | Se+/Le+ | 9.31E-02 | 6.39E-02 | 1.00E+00 |
| 6'GL | Se-/Le+ | -1.31E-01 | 4.87E-01 | 1.00E+00 |
| 6'GL | Se+/Le- | -1.31E-01 | 2.63E-01 | 1.00E+00 |
| 6'GL | Se+/Le+ | -1.84E-01 | 8.40E-03 | 3.61E-01 |
| 6'SL | Se-/Le+ | -9.60E-01 | 4.17E-05 | 2.34E-03 |
| 6'SL | Se+/Le- | -8.88E-01 | 1.18E-03 | 5.91E-02 |
| 6'SL | Se+/Le+ | -1.05E+00 | 0.00E+00 | 0.00E+00 |
| A-tetra | Se-/Le+ | 0.00E+00 | NaN | NaN |
| A-tetra | Se+/Le- | 1.98E+00 | 1.56E-01 | 1.00E+00 |
| A-tetra | Se+/Le+ | 3.47E-01 | 5.97E-01 | 1.00E+00 |
| DFLNHa | Se-/Le+ | 0.16823612 | 7.70E-01 | 1.00E+00 |
| DFLNHa | Se+/Le- | -0.5058005 | 3.15E-03 | 1.42E-01 |
| DFLNHa | Se+/Le+ | -0.7766742 | 1.78E-15 | 1.14E-13 |
| DSLNT | Se-/Le+ | -0.1841258 | 2.44E-01 | 1.00E+00 |
| DSLNT | Se+/Le- | 0.09116078 | 6.35E-01 | 1.00E+00 |
| DSLNT | Se+/Le+ | -0.1175192 | 8.08E-02 | 1.00E+00 |
| LDFT | Se-/Le+ | 0.54930614 | 1.32E-01 | 1.00E+00 |
| LDFT | Se+/Le- | 0.11640273 | 6.07E-01 | 1.00E+00 |
| LDFT | Se+/Le+ | 0.02366974 | 6.95E-01 | 1.00E+00 |
| LNDFH-I | Se-/Le+ | 0 | 1.00E+00 | 1.00E+00 |
| LNDFH-I | Se+/Le- | 0 | 1.00E+00 | 1.00E+00 |
| LNDFH-I | Se+/Le+ | -0.212474 | 1.36E-03 | 6.52E-02 |
| LNFP-I | Se-/Le+ | 0 | NaN | NaN |
| LNFP-I | Se+/Le- | 0.19570997 | 2.77E-01 | 1.00E+00 |
| LNFP-I | Se+/Le+ | -0.4079282 | 3.55E-07 | 2.13E-05 |
| LNFP-II | Se-/Le+ | -0.2907111 | 4.70E-02 | 1.00E+00 |
| LNFP-II | Se+/Le- | 0 | 1.00E+00 | 1.00E+00 |
| LNFP-II | Se+/Le+ | -0.0671894 | 4.59E-01 | 1.00E+00 |
| LNFP-III | Se-/Le+ | -0.2840417 | 1.22E-02 | 5.12E-01 |
| LNFP-III | Se+/Le- | -0.1140023 | 4.87E-01 | 1.00E+00 |
| LNFP-III | Se+/Le+ | -0.2026825 | 1.52E-07 | 9.25E-06 |
| LNFP-V | Se-/Le+ | -0.2318016 | 4.20E-02 | 1.00E+00 |
| LNFP-V | Se+/Le- | 0.10919929 | 5.47E-01 | 1.00E+00 |
| LNFP-V | Se+/Le+ | -0.1335314 | 6.17E-02 | 1.00E+00 |
| LNH-a | Se-/Le+ | -0.3285041 | 1.36E-01 | 1.00E+00 |
| LNH-a | Se+/Le- | -0.5705449 | 5.20E-05 | 2.86E-03 |
| LNH-a | Se+/Le+ | -0.5798185 | 1.15E-05 | 6.56E-04 |
| Hex4 HexNAc2 | Se-/Le+ | -0.3465736 | 3.80E-01 | 1.00E+00 |
| Hex4 HexNAc2 | Se+/Le- | -0.4054651 | 3.70E-01 | 1.00E+00 |
| Hex4 HexNAc2 | Se+/Le+ | -0.6931472 | 8.85E-07 | 5.22E-05 |
| LNnDFH | Se-/Le+ | 1.33154385 | 5.23E-01 | 1.00E+00 |
| LNnDFH | Se+/Le- | -0.4436516 | 3.58E-01 | 1.00E+00 |
| LNnDFH | Se+/Le+ | 0.37037379 | 6.86E-04 | 3.57E-02 |
| LNnFP-V | Se-/Le+ | -0.4795731 | 6.84E-05 | 3.70E-03 |
| LNnFP-V | Se+/Le- | 0.25541281 | 2.97E-01 | 1.00E+00 |
| LNnFP-V | Se+/Le+ | -0.4700036 | 9.95E-14 | 6.27E-12 |
| LNnT | Se-/Le+ | -0.5172565 | 3.85E-02 | 1.00E+00 |
| LNnT | Se+/Le- | -0.3007542 | 2.50E-01 | 1.00E+00 |
| LNnT | Se+/Le+ | -0.6044802 | 2.57E-08 | 1.59E-06 |
| LNT | Se-/Le+ | -0.5184713 | 2.72E-02 | 1.00E+00 |
| LNT | Se+/Le- | 0.02162499 | 9.32E-01 | 1.00E+00 |
| LNT | Se+/Le+ | -0.2834563 | 4.72E-04 | 2.50E-02 |
| LSTc | Se-/Le+ | -1.2685113 | 3.09E-06 | 1.79E-04 |
| LSTc | Se+/Le- | -1.3523928 | 1.19E-03 | 5.91E-02 |
| LSTc | Se+/Le+ | -1.2628643 | 0.00E+00 | 0.00E+00 |
| MFLNH-III | Se-/Le+ | -0.8640218 | 4.81E-03 | 2.12E-01 |
| MFLNH-III | Se+/Le- | -0.7624403 | 3.08E-03 | 1.42E-01 |
| MFLNH-III | Se+/Le+ | -0.8357367 | 0.00E+00 | 0.00E+00 |

**Supp table III**. Description of the models used to predict the milk groups.

| **Algorithms** | **Lower Accuracy** | **Accuracy** | **Upper**  **Accuracy** | **Accuracy**  **PValue** | | **Kappa** | **Lower**  **Recall** | **Recall** | **Upper**  **Recall** |
| --- | --- | --- | --- | --- | --- | --- | --- | --- | --- |
| mlp | 0.885 | 0.978 | 0.999 | | 0.001 | 0.938 | 0.973 | 1 | 1 |
| mlpML | 0.885 | 0.978 | 0.999 | | 0.001 | 0.938 | 0.973 | 1 | 1 |
| mlpWeightDecay | 0.885 | 0.978 | 0.999 | | 0.001 | 0.938 | 0.973 | 1 | 1 |
| multinom | 0.885 | 0.978 | 0.999 | | 0.001 | 0.938 | 0.973 | 1 | 1 |
| nnet | 0.885 | 0.978 | 0.999 | | 0.001 | 0.938 | 0.973 | 1 | 1 |
| svmLinear | 0.885 | 0.978 | 0.999 | | 0.001 | 0.938 | 0.973 | 1 | 1 |
| avNNet | 0.852 | 0.957 | 0.995 | | 0.003 | 0.881 | 0.946 | 1 | 1 |
| lda | 0.852 | 0.957 | 0.995 | | 0.003 | 0.881 | 0.946 | 1 | 1 |
| lda2 | 0.852 | 0.957 | 0.995 | | 0.003 | 0.881 | 0.946 | 1 | 1 |
| pcaNNet | 0.852 | 0.957 | 0.995 | | 0.003 | 0.881 | 0.946 | 1 | 1 |
| pda | 0.852 | 0.957 | 0.995 | | 0.003 | 0.881 | 0.946 | 1 | 1 |
| RFlda | 0.852 | 0.957 | 0.995 | | 0.003 | 0.881 | 0.946 | 1 | 1 |
| CSimca | 0.737 | 0.87 | 0.951 | | 0.178 | 0.693 | 0.838 | 1 | 1 |
| lssvmPoly | 0.764 | 0.891 | 0.964 | | 0.091 | 0.724 | 0.75 | 0.892 | 1 |
| AdaBag | 0.821 | 0.935 | 0.986 | | 0.013 | 0.813 | 0.75 | 0.946 | 1 |
| AdaBoost.M1 | 0.821 | 0.935 | 0.986 | | 0.013 | 0.813 | 0.75 | 0.946 | 1 |
| C5.0 | 0.821 | 0.935 | 0.986 | | 0.013 | 0.813 | 0.75 | 0.946 | 1 |
| C5.0Rules | 0.821 | 0.935 | 0.986 | | 0.013 | 0.813 | 0.75 | 0.946 | 1 |
| C5.0Tree | 0.821 | 0.935 | 0.986 | | 0.013 | 0.813 | 0.75 | 0.946 | 1 |
| hda | 0.821 | 0.935 | 0.986 | | 0.013 | 0.813 | 0.75 | 0.946 | 1 |
| LogitBoost | 0.821 | 0.935 | 0.986 | | 0.013 | 0.813 | 0.75 | 0.946 | 1 |
| lssvmRadial | 0.821 | 0.935 | 0.986 | | 0.013 | 0.821 | 0.75 | 0.946 | 1 |
| parRF | 0.821 | 0.935 | 0.986 | | 0.013 | 0.813 | 0.75 | 0.946 | 1 |
| rflda | 0.821 | 0.935 | 0.986 | | 0.013 | 0.813 | 0.75 | 0.946 | 1 |
| xgbTree | 0.821 | 0.935 | 0.986 | | 0.013 | 0.813 | 0.75 | 0.946 | 1 |
| kknn | 0.852 | 0.957 | 0.995 | | 0.003 | 0.87 | 0.75 | 0.973 | 1 |
| Mlda | 0.852 | 0.957 | 0.995 | | 0.003 | 0.87 | 0.75 | 0.973 | 1 |
| rf | 0.852 | 0.957 | 0.995 | | 0.003 | 0.87 | 0.75 | 0.973 | 1 |
| gaussprLinear | 0.612 | 0.761 | 0.874 | | 0.825 | 0.525 | 0.703 | 1 | 1 |
| gaussprRadial | 0.821 | 0.935 | 0.986 | | 0.013 | 0.795 | 0.5 | 0.973 | 1 |
| kernelpls | 0.737 | 0.87 | 0.951 | | 0.178 | 0.521 | 0.4 | 0.5 | 0.973 |
| sparseLDA | 0.309 | 0.457 | 0.61 | | 1 | 0.242 | 0.324 | 1 | 1 |
| mlpWeightDecayML | 0.792 | 0.913 | 0.976 | | 0.039 | 0.712 | 0.25 | 0.973 | 1 |
| naive_bayes | 0.792 | 0.913 | 0.976 | | 0.039 | 0.712 | 0.25 | 0.973 | 1 |
| nb | 0.792 | 0.913 | 0.976 | | 0.039 | 0.712 | 0.25 | 0.973 | 1 |
| gam | 0.094 | 0.196 | 0.339 | | 1 | 0.116 | 0 | 1 | 1 |
| gamLoess | 0.094 | 0.196 | 0.339 | | 1 | 0.116 | 0 | 1 | 1 |
| gamSpline | 0.094 | 0.196 | 0.339 | | 1 | 0.116 | 0 | 1 | 1 |
| ownn | 0.094 | 0.196 | 0.339 | | 1 | 0.116 | 0 | 1 | 1 |
| ORFlog | 0.078 | 0.174 | 0.314 | | 1 | 0.075 | 0 | 0.75 | 1 |
| ORFpls | 0.078 | 0.174 | 0.314 | | 1 | 0.075 | 0 | 0.75 | 1 |
| ORFridge | 0.078 | 0.174 | 0.314 | | 1 | 0.078 | 0 | 0.75 | 1 |
| ORFsvm | 0.078 | 0.174 | 0.314 | | 1 | 0.075 | 0 | 0.75 | 1 |
| evtree | 0.764 | 0.891 | 0.964 | | 0.091 | 0.62 | 0 | 0.973 | 1 |
| gpls | 0.359 | 0.833 | 0.996 | | 0.737 | 0 | NA | NA | NA |
